# Effect of Time of Daily Data Collection on the Calculation of Catheter-associated Urinary Tract Infection Rates

**DOI:** 10.1097/pq9.0000000000000466

**Published:** 2021-08-26

**Authors:** Lane F. Donnelly, Matthew Wood, Ling Loh, Natasa Tekic, Andrew Y. Shin, David Scheinker

**Affiliations:** From the * Lucile Packard Children’s Hospital—Stanford, Stanford Children’s Health, Palo Alto, CA; † Department of Pediatrics, Stanford University, School of Medicine, Palo Alto, CA; ‡ Department of Radiology, Stanford University, School of Medicine, Palo Alto, CA

## Abstract

**Introduction:**

According to the *National Healthcare Safety Network* (NHSN) definitions for Catheter-associated urinary tract infections (CAUTI) rates, determination of the number of urinary catheter days must occur by calculating the number of catheters in place “for each day of the month, at the same time of day” but does not define at what time of day this occurs. The purpose of this review was to determine if a data collection time of 11 am would yield a greater collection of urinary catheter days than that done at midnight.

**Methods:**

During a 20-month period, the number of urinary catheter days was calculated using once-a-day electronic measurements to identify a urinary catheter presence. We used data collected at 11 am and collected at midnight (our historic default) in comparing the calculated urinary catheter days and resultant CAUTI rates.

**Results:**

There were 7,548 patients who had a urinary tract catheter. The number of urinary catheter days captured using the 11 am collection time was 15,425, and using the midnight collection time was 10,234, resulting in a 50.7% increase. The CAUTI rate per 1,000 urinary catheter days calculated using the 11 am collection method was 0.58, and using the midnight collection method was 0.88, a reduced CAUTI rate of 33.6%.

**Conclusion:**

The data collection time can significantly impact the calculation of urinary catheter days and on calculated CAUTI rates. Variations in how healthcare systems define their denominator per current *National Healthcare Safety Network* policy may result in significant differences in reported rates.

## INTRODUCTION

Hospital-acquired conditions (HACs) are a significant cause of morbidity, mortality, and unnecessary expense in providing healthcare.^1–8^ Because of this, there have been multiple local and national efforts to reduce the frequency of HACs.^3–8^ HAC rates are tracked, reported to multiple sources, and have been tied to reimbursement in some cases by the *Centers for Medicare & Medicaid Services* (CMS).^9–14^ HAC rates are one of the major indicators by which healthcare systems are judged. All of these factors make HAC rate calculations important.

In calculating the rate of each HAC, the numerator is the number of occurrences of that specific HAC during the time period in question. For HACs related to a line or catheter, the denominator is the number of days that the catheter or line is in place.^1,2^ According to *National Healthcare Safety Network* (NHSN) definitions, the calculation of the number of line or catheter days must occur by one of three mechanisms: (1) a once-a-day count at a fixed time for all patients with a particular line or catheter; (2) a sampling-based approximation to the once-a-day count; (3) an electronic count that may be used “after a validation of a minimum three consecutive months proves the data to be within 5% (±) of the manually collected once-a-day counts.”^1,2,15^ For the once-a-day count at a fixed time, NHSN defines the calculation of the number of lines or catheters in place must occur “for each day of the month, at the same time of day” but does not define at what time of day this should occur.^1,2^ Using the once-a-day method, our data extraction process to obtain data about line or catheter days from the electronic health record has historically been conducted at midnight. Through informal communication with our colleagues at other children’s hospitals, many organizations use midnight as the default time when such data is collected. Regarding catheter-associated blood-stream infection (CLABSI) rates, a previous publication has shown that the time of day at which the data is collected can significantly affect the capture of line days and thus the calculated CLABSI rate.^15^

Catheter-associated urinary tract infections (CAUTIs) are a common HAC.^7^ CAUTIs are associated with increased length of hospitalization (2.4 days) as well as increased hospital charges ($7,200).^8^ As is the case with other HACs, the NHSN definition for urinary catheter days specifies the time of data collection must be at the same time each day but does not prescribe what time of day that should occur. Our healthcare system has historically used the default time of data collection for urinary catheter days as midnight. Based on published experience^15^ and our preliminary data evaluations, the time of day that seemed to capture the most urinary catheter days is sometime in the late morning. The purpose of this review was to determine if a data collection time of 11 am would yield a greater and more representative collection of urinary catheter days than that done at midnight.

## METHODS

A single-center retrospective review of urinary catheter days was performed as part of a quality improvement project to better understand and improve our CAUTI rates at a quaternary children’s hospital. This project met criteria for a quality improvement project, was not considered human subjects research, and as a result was exempt from review by our Institutional Review Board.

Urinary catheter days were determined based on data from 20 months (January 1, 2019 through August 31, 2020). The number of urinary catheter days was calculated using once-a-day electronic measurements to identify a urinary catheter’s presence or absence. We compared data collected at 11 am to that obtained at midnight. The number of urinary catheter days was compared between the 2 collection time methods, and the difference and % difference was calculated.

To assess the statistical variability of this data, we evaluated the number of urinary catheter days for each individual patient encountered who at a point in time had a urinary catheter. The mean and SD of the difference in identified urinary catheter days as counted at 11 am versus midnight across patients was calculated.

The NHSN definition for CAUTI was utilized.^1^ Based on the number of CAUTIs encountered during the study period, the CAUTI rates were compared using the urinary catheter days calculated using the 11 am and midnight collection methods. The formula used to calculate the CAUTI rate per 1,000 urinary catheter line days was the number of CAUTIs observed/urinary catheter days X 1,000.

## RESULTS

The number of urinary catheter days captured using the 11 am collection time was 15,425, and using the midnight collection time was 10,234 (Table 1). The additional 5,191 urinary catheter days identified using the 11 am collection time represents a 50.7% increase: 95% confidence interval [5096, 5286].

**Table 1.** Difference in Urinary Catheter Days and CAUTI Rates per 1,000 Urinary Catheter Days between the 11 am and Midnight Data Collection Times

Time of Day—Data Capture	Urinary Catheter Days	CAUTI Rate
11 am	15,425	0.58
Midnight	10,234	0.88
Difference	5191	0.30
% Difference	50.7%	33.6%

There were 7,548 patients with a urinary tract catheter at some point during the study period. For every patient, the number of urinary catheter days was equal or greater when measured using the 11 am method as compared to the midnight measure. The mean number of additional urinary catheter days per patient identified at 11 am versus midnight was 0.69 days (SD 0.56). The maximal number of additional urinary catheter days identified per individual patient at 11 am versus midnight was 5, and the minimum was 0. One patient had 5 additional urinary catheter days identified on the 11 am count. This was a long-term inpatient who had 5 different occurrences of having different urinary catheters in place on different days. For each occurrence, the urinary catheter was in place and captured on the 11 am data collection but was not present at midnight.

There were 9 CAUTIs during the data collection period. The CAUTI rate calculated using the 11 am collection method was 0.58, and using the midnight collection method was 0.88 (Table 1). Thus, using the 11 am collection time resulted in the collection of additional urinary catheter days that reduced the CAUTI rate by 33.6%.

## DISCUSSION

The genesis for this initiative occurred when working on a CLABSI rate reduction initiative.^5^ For that initiative, we had pulled information from our electronic health record, a large dataset encompassing 4 years of central line and associated blood stream infection data. During that analysis, the issue that for the once-a-day data collection technique, the data collection time could significantly affect the line days count and the subsequent concomitant CLABSI rate. This issue was not addressed by NSHN definitions.^1,2^ A 10 am data collection resulted in a 4%–6% decrease in the calculated CLABSI rate compared to a 7 pm collection time.^15^

This realization led us to work with our in-house quality data analytics team to assess whether moving our historical midnight collection time for central line days to another time of day would have an effect on our CLABSI rate. Our findings were more modest than those seen with analysis of the 4-year data collection. After testing various times of data collection, moving to a data collection time of 11 am yielded the greatest change, but the decrease was less than 1%.

From a practical standpoint, it made the most sense to perform the once-a-day data extraction from the electronic health record at the same time each day for all hospital-acquired conditions with associated line or catheter days. In other words, it would be impractical, and perhaps disingenuous, to use various and different times of day to calculate the central line days compared to urinary catheter days and other key performance indicators. As we considered the effects of changing the time of day for the data collection on different indicators, it led us to examine the effect on urinary catheter days and CAUTI rates.

Our analysis showed that moving the time of data collection for urinary catheter presence from midnight to 11 am resulted in an increased capture of urinary catheter days by greater than 50%, which translated into a reduction of the calculated CAUTI rate by 33.6%. This change in the data collection time had a significantly more profound effect on the CAUTI rate than was previously described for CLABSI rates.^15^ This is likely related to the fact that urinary catheters are often in place for shorter periods than are central venous catheters and, therefore, more sensitive to changes in the data collection time of day.

One might think that there could be patients who had a urinary catheter in place at midnight that was not in place at 11 am, who would be captured in a midnight count only, and that such incidents could lower the urinary catheter days captured at 11 am. However, in every individual patient of the 7,548 evaluated, the counted urinary catheter days were greater using the 11 am as compared to the midnight data collection. Of course, no specific time for data collection would be perfect. For example, a patient who had a urinary catheter placed at 8 am and removed at 10 am would not be captured in either a 11 am or midnight process. There are clear reasons why collecting data at 11 am yields a greater capture of urinary catheter presence than when performed at midnight. Many urinary catheters that are placed for early morning surgery or other procedures are likely still in place and counted in an 11 am data collection but have been removed in the afternoon or early evening and therefore do not appear in a midnight count. Likewise, a significant number of patients are discharged after 11 am, in the afternoon or evening, and might have a urinary catheter in place and counted at 11 am but are not present for a data collection performed at midnight.

Given the significance of CAUTI rates and other HAC rates as a marker of safe patient care, external benchmarking of healthcare systems,^9^ and reimbursement,^10–14^ we believe that it is important the denominator used in the calculation of those rates reflect the capture of all pertinent catheters. Variations in how healthcare systems using the once-a-day data collection method choose the time of day to collect that data under the current NHSN policy may result in significant differences in reported rates from organization to organization.

Historically, hospital volume, utilization, and bed occupancy have been measured by evaluating the number of patients in beds at midnight, “the midnight census.” This may historically relate to a time of day in which patients were asleep in their rooms, not traveling to other parts of the hospital for procedures, not being discharged, and therefore easy to manually count. Despite limitations raised about midnight data collection^16–18^ and the electronic health records allowing for such information to be generated at any time of day, the use of the “midnight census” remains common.^16–18^ The use of the “midnight census” has led many healthcare systems to use midnight as the default time, not only to collect the patient census, but also for data collection for other metrics such as the central line and urinary catheter days. This potentially significantly undercounts the number of urinary catheter days, and therefore artificially inflates associated CAUTI rates. We strongly believe the NHSN should consider revising their definitions for “once-a-day data collection methodology” to a standard time of day for urinary catheter days and potentially other HACs. In the meantime, we advocate that healthcare systems should study and select the collection time that optimizes the capture of urinary catheter days. In our experience and that previously published, that optimal time is late morning. We are changing our data capture time for line and catheter days to 11 am for all pertinent HACs.

This study has several limitations. First, it is a single-center study. Second, related to variability in number of post-procedural urinary catheters between children’s hospitals, the optimal time of day to optimally capture urinary catheter days may not be universal. Third, the study has only a small number of CAUTIs. However, the data analyzed was from 20 months and because CAUTI is a relatively rare event, there were only nine CAUTIs during the study period. However, this study had sufficient catheter days to easily make the point that it is the denominator (catheter days) that is key to ensure comparable CAUTI rates (intrainstitutional and interinstitutional). The study also only evaluated one of the 3 methods by which NSHN definitions allow for urinary catheter day calculations—the once-a-day method. The other 2 methods—sampling-based approximation and electronic counts—were not evaluated.^1,2,9^ Another limitation is that although moving the time of data collection may increase the number of identified urinary catheters, some of those catheters are placed for surgery in the morning and are taken out in the afternoon. They may not represent a “full” urinary catheter day, as they are often likely not present for a full 24 hours. Therefore, although the number of urinary catheters captured is greater, this may not fully represent the CAUTI risk, as it is well known that the duration of catheter presence is risk factor for CAUTI development. Finally, we relied on electronic documentation of catheter presence. There could be inaccuracies in what was or was not documented.

In conclusion, for the once-a-day catheter presence method, the data collection time can significantly impact identified urinary catheter days and associated CAUTI rates. Variations in how healthcare systems define their denominator under the current NHSN policy may significantly differ in reported rates, making the comparison of rates between healthcare organizations less meaningful.

## DISCLOSURE

The authors have no financial interest to declare in relation to the content of this article.
